# l-carnosine enhanced reproductive potential of the *Saccharomyces cerevisiae* yeast growing on medium containing glucose as a source of carbon

**DOI:** 10.1007/s10522-016-9645-9

**Published:** 2016-04-04

**Authors:** Magdalena Kwolek-Mirek, Mateusz Molon, Pawel Kaszycki, Renata Zadrag-Tecza

**Affiliations:** Department of Biochemistry and Cell Biology, Faculty of Biology and Agriculture, University of Rzeszow, Zelwerowicza 4, 35-601 Rzeszow, Poland; Unit of Biochemistry, Institute of Plant Biology and Biotechnology, Faculty of Biotechnology and Horticulture, University of Agriculture in Krakow, al. 29 Listopada 54, 31-425 Kraków, Poland

**Keywords:** l-carnosine, Lifespan, Reproductive potential, Yeast, Mitochondrial membrane potential, ATP

## Abstract

Carnosine is an endogenous dipeptide composed of β-alanine and l-histidine, which occurs in vertebrates, including humans. It has a number of favorable properties including buffering, chelating, antioxidant, anti-glycation and anti-aging activities. In our study we used the *Saccharomyces cerevisiae* yeast as a model organism to examine the impact of l-carnosine on the cell lifespan. We demonstrated that l-carnosine slowed down the growth and decreased the metabolic activity of cells as well as prolonged their generation time. On the other hand, it allowed for enhancement of the yeast reproductive potential and extended its reproductive lifespan. These changes may be a result of the reduced mitochondrial membrane potential and decreased ATP content in the yeast cells. However, due to reduction of the post-reproductive lifespan, l-carnosine did not have an influence on the total lifespan of yeast. In conclusion, l-carnosine does not extend the total lifespan of *S. cerevisiae* but rather it increases the yeast’s reproductive capacity by increasing the number of daughter cells produced.

## Introduction

Carnosine (β-alanyl-l-histidine) is a water-soluble dipeptide which occurs naturally in the millimolar range in mammals, including humans. The highest concentrations of carnosine are observed in the skeletal muscle tissue, central nervous system and cardiac muscle, with lower concentrations found in stomach, liver and kidney. It can also be found in muscles of fish, amphibians, reptiles and birds, but never in plants, fungi or other eukaryotes (Boldyrev et al. 2013). Carnosine has three ionisable groups: the amino group of β-alanine as well as the carboxylic group and the nitrogens of imidazole ring of l-histidine. This chemical structure of carnosine determines its properties. The nitrogen atoms of carnosine imidazole ring (p*K*_a_ = 6.72) regulate the buffering activity of the dipeptide, which is particularly important in the skeletal and cardiac muscle. Carnosine also displays the metal ion chelating activity. It can form complexes with Cu^2+^, Co^2+^, Ni^2+^, Cd^2+^ and Zn^2+^, which have a wide range of biological relevance (Hill and Blikslager 2012; Mizuno et al. 2015; Mozdzan et al. 2005). Furthermore, carnosine is well-known for its antioxidant properties. It has both the ability to directly scavenge reactive oxygen species such as peroxynitrite and hypochlorite, and increase the content and/or regeneration of enzymatic and non-enzymatic antioxidants (Fontana et al. 2002; Hipkiss et al. 1998b; Kim et al. 2011; Klebanov et al. 1997). It was also confirmed that carnosine participates in prevention of the formation of advanced lipoxidation end-products (ALEs) and advanced glycation end-products (AGEs). Carnosine is not only able to prevent protein carbonylation but can also react directly with protein carbonyl groups, producing protein–carbonyl–carnosine adducts that prevent cross-linking to other unmodified proteins (Aldini et al. 2005; Brownson and Hipkiss 2000; Hipkiss et al. 1998a; Xie et al. 2013).

Under physiological conditions, the endogenous carnosine plays a crucial role in the skeletal and cardiac muscle as well as the neuronal tissue. In turn, the exogenous carnosine is considered a potential therapeutic agent for many diseases such as diabetes, ischemia/reperfusion damage, ocular diseases and neurological disorders (Alzheimer and Parkinson disease, schizophrenia and autistic spectrum disorders) (Aldini et al. 2005; Bellia et al. 2011; Boldyrev et al. 2013; Hipkiss 2007). Carnosine also reveals anti-cancer and anti-aging activities. It inhibits selectively the growth of transformed cell lines and tumour cells by suppressing cellular ATP generation (Iovine et al. 2012; Renner et al. 2010; Shen et al. 2014). In contrast, McFerland and Holliday (1994) demonstrated that carnosine could increase the lifespan as well as chronological age of cultured human diploid fibroblasts. In addition, it was found to rejuvenate already senescent cells giving them a more juvenile appearance (Holliday and McFarland 2000). Significant increase in the lifespan was also reported in peripheral blood derived human CD4+ T cell clones after long-term culture with carnosine (Hyland et al. 2000). The anti-aging effect of carnosine has been described both for the cell lines and animal models. The study by Boldyrev group reported that carnosine supplemented to a standard diet attenuated the development of senile features and increased the lifespan in senescence-accelerated mice (Boldyrev et al. 1999; Gallant et al. 2000). It proved to increase significantly the number of spermatogonia and Sertoli cells in mice prone to accelerated aging (Gopko et al. 2005), and furthermore, extended the lifespan of male *Drosophila melanogaster* flies (Yuneva et al. 2002) and *Brachionus manjavacas* rotifers (Snell et al. 2012).

Studies of the influence of various chemical factors on the cellular or organismal level have been conducted on a wide range of model organisms. Among these, the yeast *Saccharomyces cerevisiae* is a commonly used organism to study the influence of such factors on the growth, lifespan and aging process (Krzepilko et al. 2004; Lam et al. 2010; Wu et al. 2014). This yeast has also been used to study the effect of carnosine. Cartwright et al. (2012) showed that carnosine exhibited either inhibitory, or stimulatory effects on yeast cells, depending on the carbon source in the growth medium.

The aim of this study was to investigate the effect of l-carnosine on the rate of growth, reproductive potential, lifespan and metabolic activity of *S. cerevisiae* cells cultivated in the medium supplemented with glucose as a source of carbon. We also tested the cellular ATP content and mitochondrial membrane potential as affected by the presence of the studied dipeptide.

## Materials and methods

### Yeast strain, media and growth conditions

In the study a wild-type strain BY4742 MATα *his3Δ1 leu2Δ0 lys2Δ0 ura3Δ0* (EUROSCARF) was used. The yeast was grown in the standard liquid YPD medium (1 % Yeast Extract, 1 % Yeast Bacto-Peptone, 2 % glucose) on a rotary shaker at 150 rpm, or on the solid YPD medium containing 2 % agar, at the temperature of 28 °C.

### Determination of cell growth

Liquid yeast cultures (5 × 10^6^ cells/ml in a total volume of 200 µl of cells) with or without 20 mM l-carnosine were cultivated in a Heidolph incubator 1000 at 1200 rpm at 28 °C. Their growth was monitored turbidimetrically at 600 nm in an Anthos 2010 type 17,550 microplate reader for 12 h (measured every 1 h), then after 24 and 48 h. The relative growth rate was calculated at the exponential growth phase using an appropriate formula (Hall et al. 2014). All the data represent mean values obtained in four independent experiments.

### Determination of cell lifespan

*Saccharomyces cerevisiae* cell lifespan was determined as described previously (Minois et al. 2005; Zadrag et al. 2008). Overnight yeast cultures were dropped onto the YPD plates with the solid medium containing Phloxine B at the concentration of 10 µl/ml. During the manipulation, the plates were kept at 28 °C for 16 h and at 4 °C overnight. The reproductive potential (the number of buds produced), reproductive lifespan (the time during which a yeast cell is able to reproduce), post-reproductive lifespan (yeast cell life duration after the cessation of reproduction) and total lifespan (sum of the reproductive and post-reproductive lifespans) were analysed for forty single cells in each experiment. The data represent mean values from two separate experiments.

### Incubation and growth conditions

Yeast cells from the exponential phase culture were centrifuged, washed with sterile water and suspended either to the final density of 10^8^ cells/ml in 100 mM phosphate buffer with pH 7.0 containing 0.1 % glucose and 1 mM EDTA, or to the final density of 5 × 10^6^ cells/ml in the YPD medium; in both cases with or without addition of 20 mM l-carnosine. After 1, 3 and 6 h of incubation in buffer or 3, 6, 12, 24, 48 and 72 h of growth in YPD medium, the cells were pelleted by centrifugation, then washed twice with sterile water and used for further analysis.

### Assessment of mitochondrial membrane potential

The mitochondrial membrane potential was assessed with both rhodamine 123 and rhodamine B hexyl ester according to the manufacturer’s protocol (Molecular Probes). The cells after growth in the presence or absence of l-carnosine were suspended either in 50 mM citrate buffer with pH 5.0 containing 2 % glucose, or in 10 mM HEPES buffer with pH 7.4 containing 5 % glucose, for the case of rhodamine 123 and rhodamine B hexyl ester staining, respectively. Rhodamine 123 was added to the final concentration of 5 µM, and after 15 min of incubation the fluorescence of the cell suspension was measured using the TECAN Infinite 200 microplate reader at λ_ex_ = 505 nm and λ_em_ = 534 nm. The data represent mean values obtained upon three independent experiments. The mitochondrial network was visualised by fluorescence microscopy Olympus BX-51 using 100 nM rhodamine B, the fluorescent dye in which the emission is dependent on the mitochondrial membrane potential, at λ_ex_ = 555 nm and λ_em_ = 579 nm. The photographs present a typical result of a duplicate experiment.

### Assessment of the cellular ATP content

The level of ATP in the yeast cells was assessed with BacTiter-Glo™ Microbial Cell Viability Assay according to the manufacturer’s protocol (Promega). The cells after incubation or growth in the presence or absence of l-carnosine were suspended in a 100 mM phosphate buffer with pH 7.0 containing 0.1 % glucose and 1 mM EDTA. A sample of cell suspension with a density of 10^6^ cells/ml was used for determination purposes. The luminescent signal, as proportional to the amount of ATP present, was recorded after 5 min using the TECAN Infinite 200 microplate reader. The cellular ATP content was calculated from the standard curve. The data represent mean values from four independent experiments.

### Assessment of the metabolic activity

The metabolic activity of the yeast cells was assessed with FUN-1 according to the manufacturer’s protocol (Molecular Probes). The cells after incubation or growth in the presence or absence of l-carnosine were suspended in a 10 mM HEPES buffer with pH 7.2 containing 2 % glucose. The metabolic activity of cells was estimated with 0.5 µM FUN-1. The fluorescence of the cell suspension was measured after 15 min using the TECAN Infinite 200 microplate reader at λ_ex_ = 480 nm and λ_em_ = 500–650 nm. The metabolic activity of cells was expressed as a change in ratio of red (λ = 575 nm) to green (λ = 535 nm) fluorescence. The data represent mean values from four independent experiments.

### Statistical analysis

Data are presented as mean values ± standard deviation (SD). The statistical analysis was performed using the SPSS 21.0 software. The statistical significance of the differences between the means of treated sample compared to untreated control was estimated using the *t* test for independent samples. The differences between samples obtained after various time of incubation/growth with or without addition of l-carnosine were evaluated using one-way ANOVA and the Dunnet post hoc test. Homogeneity of variance was checked using Levene’s test. Values were considered significant if *p* < 0.05.

## Results

### l-carnosine slows down the growth rate of yeast cells

**Fig. 1:**
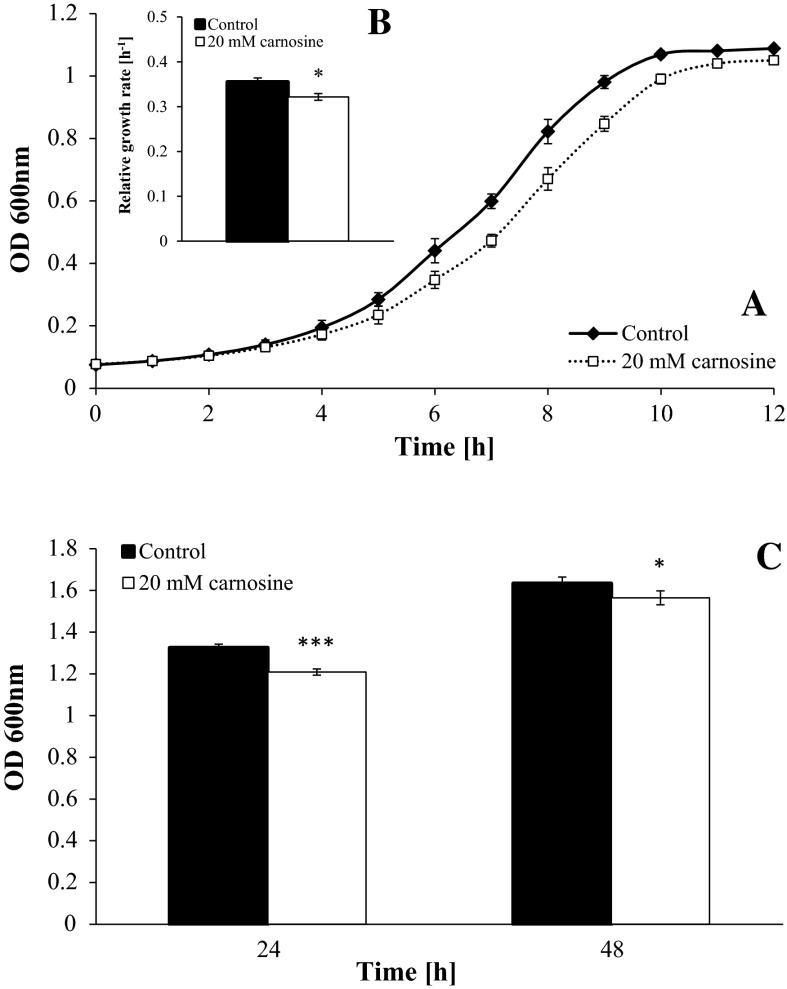
Effect of l-carnosine on growth of the BY4742 strain in liquid YPD media. Kinetics of growth was monitored turbidimetrically at 600 nm every 1 h for 12 h (**a**), and after 24 and 48 h (**c**). The relative growth rate (**b**) was calculated using appropriate formula. Data are presented as mean ± SD from four independent experiments. *p < 0.05; ***p < 0.001 as compared to the untreated control

Cartwright et al. (2012) have shown that l-carnosine decreased the growth of yeast cells in a medium containing a fermentable carbon source in a dose-dependent manner. The results in Fig. 1 confirm that the addition of 20 mM l-carnosine to the liquid medium containing 2 % glucose slowed down the growth of the BY4742 strain yeast cells. This effect was visible both for the exponential phase of growth (Fig. 1a) and after 24 and 48 h of culture (Fig. 1c). We also observed a statistically significant decrease of the relative growth rate (Fig. 1b) and an increase in the average generation time as determined based on the growth curve by approx. 9 % (data not shown) in the case of yeast cells exposed to l-carnosine, compared to the untreated control.

### l-carnosine extends the reproductive potential but not the total lifespan of yeast cells

**Fig. 2:**
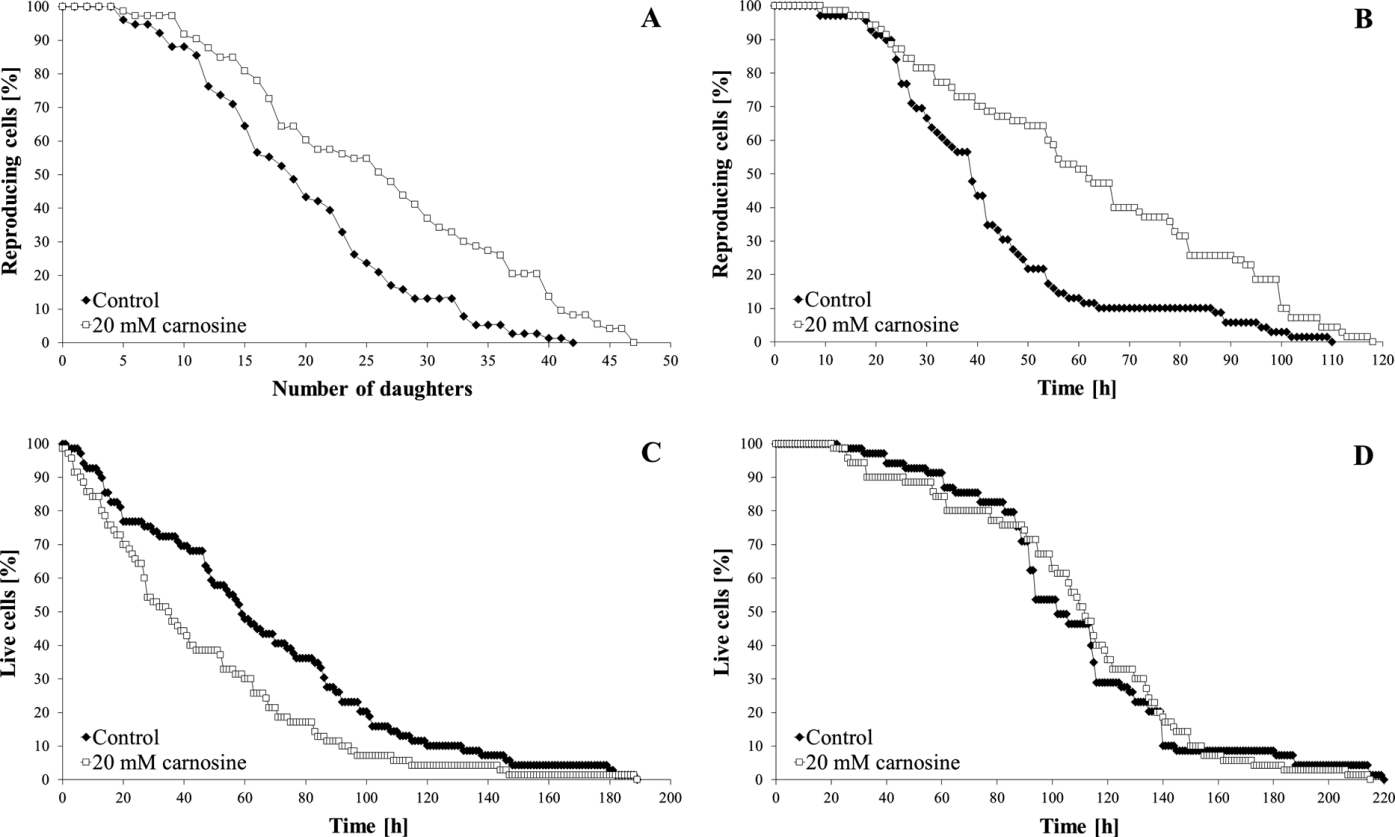
Reproductive potential (number of generations; **a**), reproductive lifespan (**b**), post-reproductive lifespan (**c**) and total lifespan (**d**) of the BY4742 strain after cultivation on solid YPD media with or without addition of 20 mM l-carnosine. Data represent mean values from two independent experiments

The reproductive potential and the total lifespan of yeast cells are not synonymous. The reproductive potential is defined as the number of daughter cells produced by a single mother cell during its life while the total lifespan is means the cell life duration including both the reproductive and post-reproductive phases. Therefore, we examined the effect of 20 mM l-carnosine on these two parameters of the BY4742 yeast. l-carnosine significantly enhanced the reproductive potential of yeast cells, increasing the average number of daughters produced from 20 to 26 (Fig. 2a; Table 1). The positive effect of l-carnosine was even more pronounced when the time-parameter was taken into account. An increase in the average generation time (determined based on the reproductive lifespan) was observed and thus the average reproductive lifespan was extended by approx. 16 and 49 %, respectively (Fig. 2b; Table 1). On the other hand, l-carnosine was found to reduce the average post-reproductive lifespan of yeast cells (during this time the cells were still alive but not able to reproduce) by approx. 46 % (Fig. 2c; Table 1). Interestingly, the total lifespan in the case of both the control conditions and the cells exposed to l-carnosine was almost the same (Fig. 2d; Table 1). These results show that l-carnosine is not able to extend the total lifespan of yeast cells; however, it can significantly influence their reproductive potential.

**Table 1:** Reproductive potential (number of daughter cells produced), reproductive lifespan, post-reproductive lifespan and total lifespan of BY4742 strain after cultivation on YPD media supplemented with 20 mM l-carnosine, control—no carnosine supplementation

	Reproductive potential	Reproductive lifespan	Post-reproductive lifespan	Total lifespan
	Number of daughter	Time (h)	Time (h)	Time (h)
Control	20 ± 8.4	42.1 ± 21.8	66 ± 45	108.1 ± 40.6
20 mM l-carnosine	25.7 ± 11.7**	62.6 ± 29.7***	45.2 ± 38.1**	107.8 ± 42.2

Data are presented as mean values ± SD obtained from all the cells tested during two independent experiments (80 cells)

** *p* < 0.01; *** *p* < 0.001 as compared to the untreated control using a *t* test

### l-carnosine decreases the cellular energy level and the metabolic activity

**Fig. 3:**
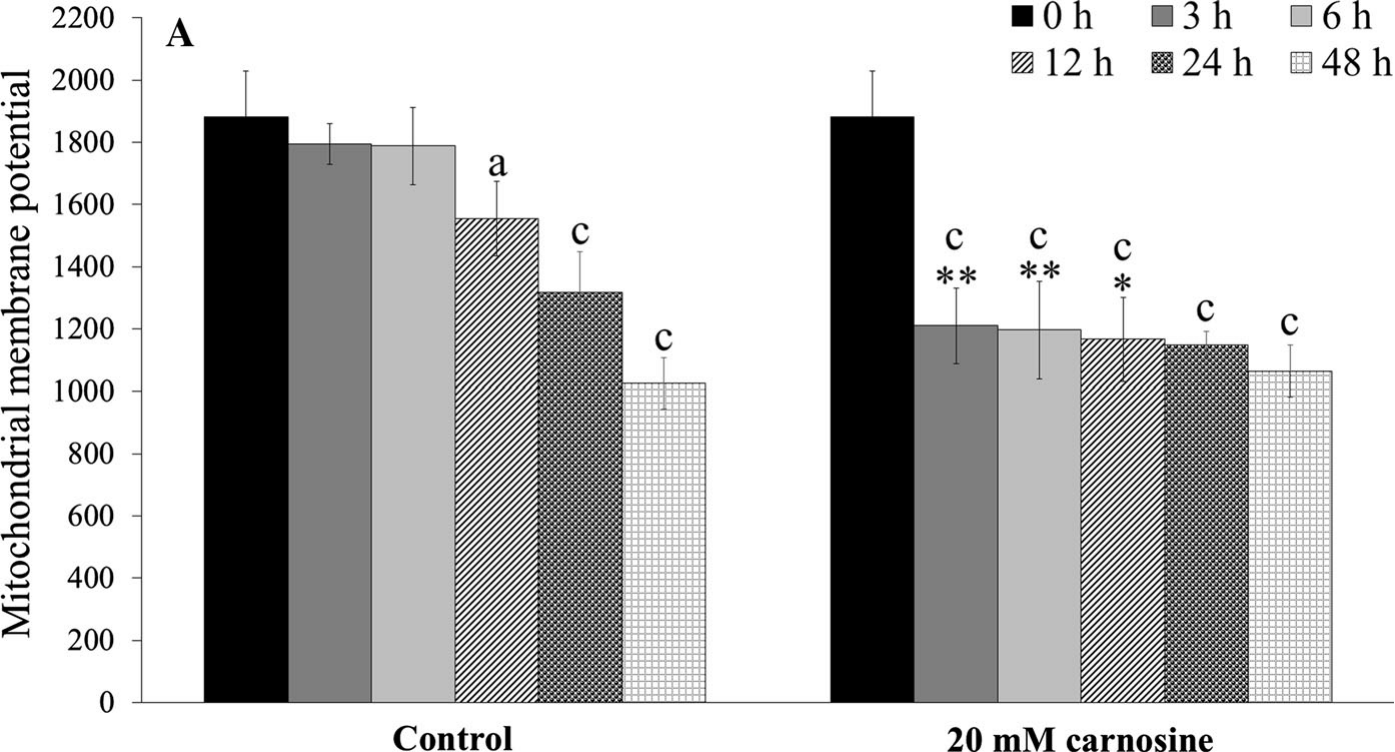

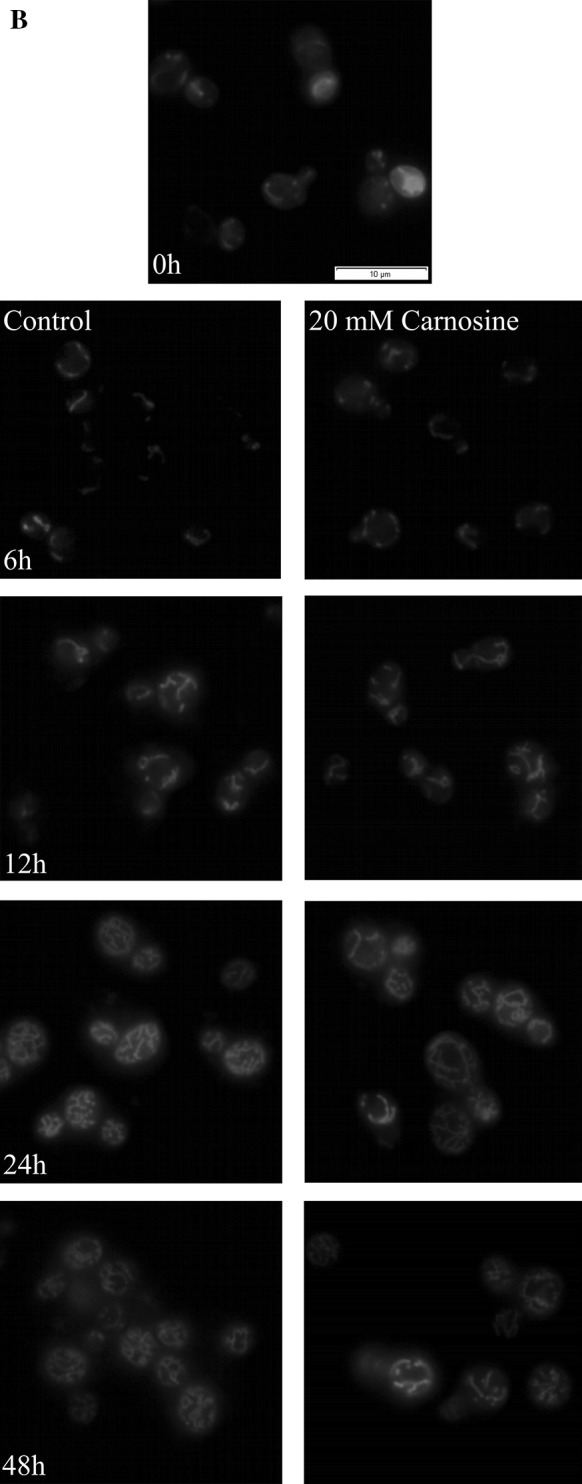
Mitochondrial membrane potential of the BY4742 yeast cells after cultivation in YPD media with or without addition of 20 mM l-carnosine. The cells were stained both with rhodamine 123 (**a**) and rhodamine B (**b**). Data are presented as mean ± SD from three independent experiments. *p < 0.05; **p < 0.01; ***p < 0.001 as compared to the untreated control. *Letters a, b and c* on the graph indicate differences between the initial and subsequent times of experiment at *p* < 0.05; *p* < 0.01; *p* < 0.001, respectively

The yeast *S. cerevisiae* receives energy from the fermentation process in the presence of fermentable carbon sources such as glucose or from the aerobic respiration in the presence of non-fermentable carbon sources such as glycerol. Therefore, mitochondria are an important cellular energy centre, and their morphology and number may change under different conditions and along with age. The effect of 20 mM l-carnosine on the mitochondrial membrane potential of the BY4742 cells was examined after short- and long-term culture. We observed that mitochondrial membrane potential after 3, 6 and 12 h of cultivation with l-carnosine remained at a relatively constant level, significantly lower than in the case of the untreated control (Fig. 3a). The level of mitochondrial membrane potential was almost the same regardless the time of cultivation (sample after 24 and 48 h) (Fig. 3a, b). Moreover, l-carnosine did not change the morphology of mitochondria and only slightly affected the development of mitochondrial network (Fig. 3b).

**Fig. 4:**
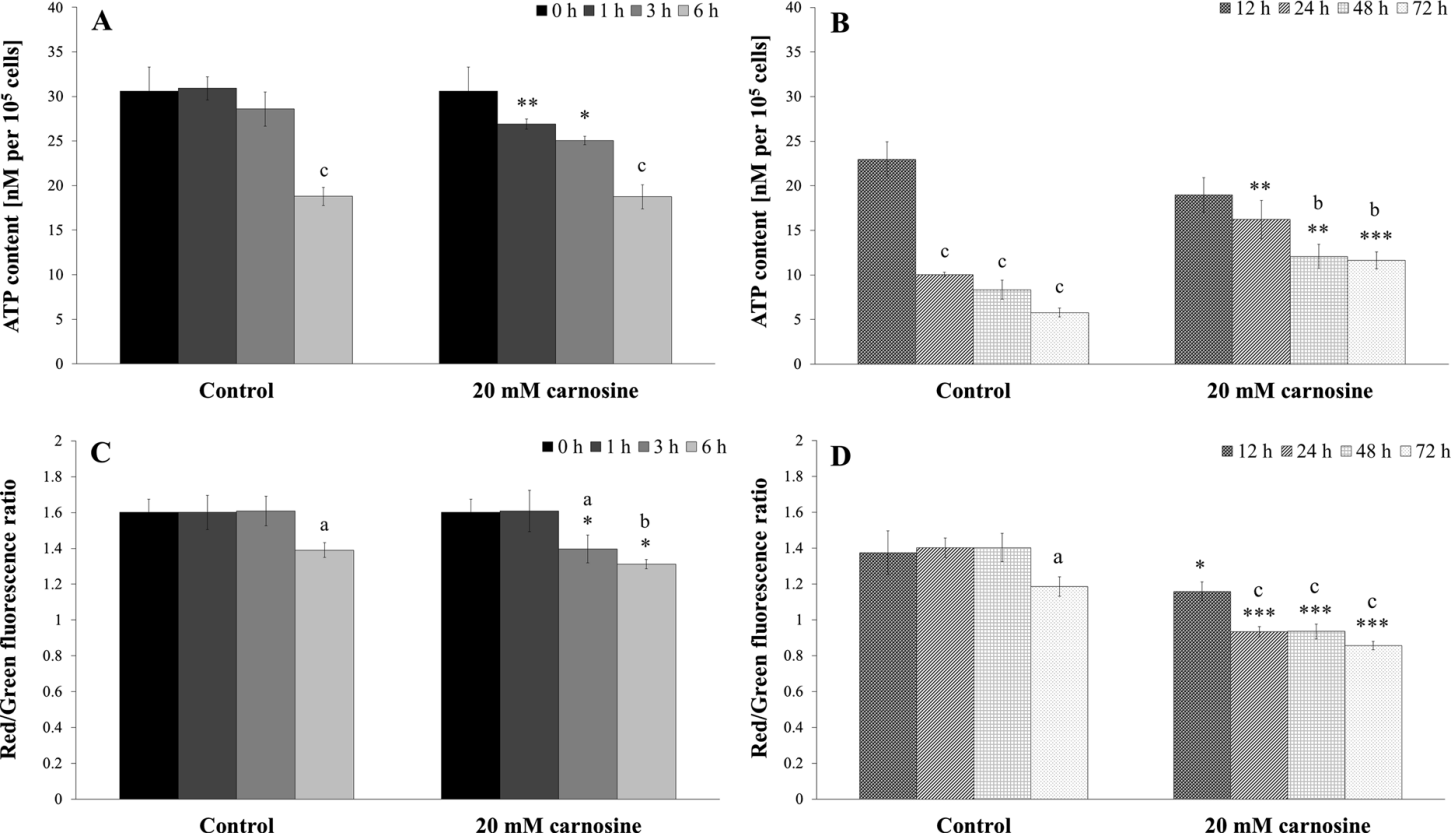
Effect of l-carnosine on ATP content and metabolic activity of the BY4742 yeast cells. Cellular ATP content (**a**, **b**) determined using BactTiter-Glo™ Microbial Cell Viability Assay and metabolic activity (**c**, **d**) using FUN-1 stain. **a**, **c** results for short time of incubation, **b**, **d** results for long-term of culture. Data are presented as mean ± SD from four independent experiments. *p < 0.05; **p < 0.01; ***p < 0.001 as compared to untreated control. *Letters a, b and c* on the graph indicate differences between the initial and subsequent times of experiment at *p* < 0.05; *p* < 0.01; *p* < 0.001, respectively

The ATP yeast cell content is directly related to the level of glucose in the medium and the mitochondrial activity. 20 mM l-carnosine decreased the level of ATP, but only after a short time of incubation/culture (Fig. 4a, b). In turn, after 24, 48 and 72 h of cultivation an opposite reaction was observed in the presence of l-carnosine: the cellular ATP content was significantly higher in comparison to the untreated control (Fig. 4b).

The cellular energy level has an impact on the vitality and viability of cells. Therefore, the effect of 20 mM l-carnosine on the metabolic activity of the BY4742 cells was determined using a FUN-1 stain. l-carnosine decreased the metabolic activity, both after a short time of incubation (3 and 6 h) and after a long-term culture (12, 24, 48 and 72 h). For each case studied these values were significantly lower compared to the untreated control (Fig. 4c, d). These results show that l-carnosine can alter the cellular energy level, thereby reducing the metabolic activity of cells.

## Discussion

The search for compounds that improve quality and prolong the time of human life has been conducted for many years in numerous laboratories around the world. Carnosine appears as one of such type of compounds with potential anti-aging properties. Previous studies show that l-carnosine could increase the lifespan as well as chronological age of human fibroblasts (Holliday and McFarland 2000; McFarland and Holliday 1994) and extend the lifespan of selected animals such as mice (Boldyrev et al. 1999; Gallant et al. 2000), *D. melanogaster* flies (Yuneva et al. 2002) and *B. manjavacas* rotifers (Snell et al. 2012). The *S. cerevisiae* yeast is commonly used as a model organism to study the influence of various factors on the growth, lifespan and aging process (Krzepilko et al. 2004; Lam et al. 2010; Wu et al. 2014). For the studies concerning carnosine, usefulness of the *S. cerevisiae* is especially important because as a fungus this yeast does not produce l-carnosine and its metabolites, which enables us to examine the effect of extracellular l-carnosine on the cells using various doses and growth conditions. *S. cerevisiae* was first subjected to the research on properties of carnosine by Cartwright et al. (2012). Their study showed that l-carnosine decreased the yeast growth in media containing fermentable carbon sources in a dose-dependent manner. This effect was more pronounced in the case of glucose than mannose, galactose or fructose. l-carnosine did not have an inhibitory effect on the growth on the contrary provoked significant increase in the growth rate of yeast in media with non-fermentable carbon sources in the presence of oxygen. The results showed that l-carnosine decreased viability of cells but only under reduced oxidative phosphorylation conditions. Our results confirm that l-carnosine slows down the growth of yeast on the YPD medium, decreases the relative growth rate and increases the generation time (Fig. 1a–c). However, the observed growth rate changes are the result of slowdown of cell reproduction cycle rather than the cell death.

Natural compounds with anti-aging properties are discovered relatively rare. Literature data indicate that l-carnosine may indeed exhibit such features (Boldyrev et al. 1999; McFarland and Holliday 1994; Snell et al. 2012; Yuneva et al. 2002). As the available references do not seem to fully confirm the case of the yeast *S. cerevisiae* (Cartwright et al. 2012; Fig. 1), we decided to investigate the effect of l-carnosine on individual cells of the mentioned species during the culture on solid YPD medium. Our study proves that 20 mM l-carnosine significantly enhanced the reproductive cell potential (the number of buds produced, which in the literature is termed as a replicative lifespan) and extended the reproductive lifespan (the time during which a yeast cell is able to reproduce) of the BY4742 strain (Fig. 2a, b; Table 1). In the literature, only few compounds were reported as having the ability to increase the reproductive potential of yeast, such as resveratrol (Howitz et al. 2003), ascorbate (Krzepilko et al. 2004), diazaborine (Steffen et al. 2008) and ibuprofen (He et al. 2014). More information can be found regarding the effects of various factors on prolonging the chronological lifespan of yeast (Georgieva et al. 2015; Nakaya et al. 2014; Rockenfeller et al. 2015; Wanke et al. 2008; Wu et al. 2014). However, one should not compare these two types of experiments since the chronological lifespan and the total lifespan are not the same. Yeast cells do not end their life immediately after the reproduction phase; therefore, an analysis of the post-reproductive phase makes it possible to determine their total lifespan. Our results indicate that 20 mM l-carnosine reduced the average post-reproductive lifespan of yeast (Fig. 2c; Table 1). It follows that the addition of l-carnosine extends the reproductive lifespan and thereby shortens the post-reproductive lifespan of yeast. The negative correlation between the post-reproductive and reproductive lifespans was presented in our previous study for a number of yeast strains (Molon et al. 2015; Zadrag-Tecza et al. 2013). Here, the total lifespan was shown to be almost the same, both for the case of the control and the cells exposed to l-carnosine (Fig. 2d; Table 1). These results prove that l-carnosine has no pro-longevity effect because it does not extend the total yeast lifespan but rather causes an increase in its reproductive potential.

Enhancing the reproductive potential of the yeast treated with l-carnosine may be associated with the regulation of energy metabolism. Previous studies report that l-carnosine inhibits the ATP production and thus reduces the proliferative capacity of cancer cells (Iovine et al. 2012; Renner et al. 2010; Shen et al. 2014). Furthermore, Cartwright et al. (2012) demonstrated that l-carnosine caused changes in the metabolic activity of the yeast grown on the fermentable carbon source. Addition of l-carnosine significantly affected the heat output profiles of the cultures, measured using on-line flow microcalorimetry, in a dose-dependent manner (Cartwright et al. 2012). Based on our work, it is clearly seen that 20 mM l-carnosine decreases mitochondrial membrane potential during the culture on YPD medium compared to the control, but only in the exponential phase of growth. The differences in the mitochondrial membrane potential are not visible in the stationary phase of growth when most of the glucose supply has been consumed and the yeast cells have transferred to the aerobic respiration (Fig. 3a). A mild decrease in the mitochondrial membrane potential is considered beneficial for cells and whole organism (Knorre and Severin 2012). This has been confirmed by the results of Barros et al. (Barros et al. 2004) with the use of low doses of protonophore dinitrophenol which caused an increase in the number of daughters produced (replicative lifespan). The proposed mechanism assumes that lowering the mitochondrial membrane potential may lead to preventing mitochondrial production of reactive oxygen species (ROS) but also may activate the retrograde response, which by transcriptional changes can result in an increase of the lifespan (Miceli et al. 2011).

We also observed a significant reduction of the ATP content in the cells after short incubation time in the presence of 20 mM l-carnosine (Fig. 4a, b). In turn, after 24, 48 and 72 h of culture we observed an opposite reaction and a significantly higher level of cellular ATP compared to the control (Fig. 4b). This effect may be a result of lower cell energy requirements in the presence of l-carnosine, its protective properties or amino acid supplementation deriving from carnosine metabolism. Such an increase in the ATP level enables the cells to increase the number of daughters produced (reproductive potential), and in terms of the time of a single reproduction cycle, to extend the reproductive lifespan (Fig. 2a, b; Table 1). The observed changes in ATP levels during prolonged culture can result from a changes in a way of energy production by yeast cells. At a high level of glucose it dominates the fermentative metabolism, which is changed into a respiration after consumption the majority of available glucose. The initial decrease in ATP level may result from the fact, that the addition of l-carnosine reduces the ATP production from glycolysis, as is demonstrated in the case of tumour cells (Renner et al. 2010). In turn the observed increase in the ATP level occurs after diauxic shift, where metabolism becomes aerobic. However, a higher level of ATP (which was still very low) made it impossible to maintain metabolic activity of the cells at a constant level (Fig. 4d). The addition of 20 mM l-carnosine decreased the metabolic activity both at the exponential and stationary growth phases as compared to the control (Fig. 4c, d). These results confirm the earlier observations of Cartwright et al. (2012).

In summary, l-carnosine can change the cellular ATP content by decreasing the intensity of glycolysis which results in a reduction of the mitochondrial membrane potential, a decrease in the metabolic activity of cells and the extended time of generation. Reduction of the energy level in the cells leads to the enhanced reproductive potential and extended reproductive lifespan of yeast. As a regulator of cell energy metabolism, l-carnosine causes an efficient increase in the reproductive capacity as typically observed in the case of caloric restriction and repression of the enhanced gluconeogenesis (Evans et al. 2010; Hachinohe et al. 2013; Lin et al. 2002; Medvedik et al. 2007; Wierman and Smith 2014). On the other hand, we must not forget that due to reduction of the post-reproductive lifespan the effect of l-carnosine on the total lifespan of yeast is not significant. l-carnosine does not extend the lifetime of a single yeast cell but can rather increase the chances for population survival by increasing the number of offspring.
